# Global Density Profile For Particle Non-Conserving One Dimensional Transport From Renormalization Group Flows

**DOI:** 10.1038/s41598-019-42011-5

**Published:** 2019-04-05

**Authors:** Sutapa Mukherji, Somendra M. Bhattacharjee

**Affiliations:** 1 Protein Chemistry and Technology, CSIR - Central Food Technological Research Institute, Mysore 570020, India; 2 Institute of Physics, Bhubaneswar 751005, India

## Abstract

The totally asymmetric simple exclusion process along with particle adsorption and evaporation kinetics is a model of boundary-induced nonequilibrium phase transition. In the continuum limit, the average particle density across the system is described by a singular differential equation involving multiple scales which lead to the formation of boundary layers (BL) or shocks. A renormalization group analysis is developed here by using the location and the width of the BL as the renormalization parameters. It not only allows us to cure the large distance divergences in the perturbative solution for the BL but also generates, from the BL solution, an analytical form for the global density profile. The predicted scaling form is checked against numerical solutions for finite systems.

## Introduction

The totally asymmetric simple exclusion process (TASEP) is a model for boundary-induced nonequilibrium phase transitions^1–3^, though it had its genesis in modeling polymerization on biopolymeric templates^4,5^. In this open, driven system, particles, representing biomolecules, hop in a specific direction on a one-dimensional lattice, obeying a mutual exclusion rule forbidding double occupancy of any site. The rates of injection and withdrawal of particles at the boundaries are the drives necessary to maintain the system in a nonequilibrium steady state and they determine the bulk properties, for example, the average particle density in the bulk, in the steady-state. Unlike equilibrium systems, there is a bulk-boundary duality (BBD) and the bulk transitions are completely encoded in thin boundary layers (BL) of the particle density. BLs are not just microscopic details because they survive the continuum limit which washes out some small-scale details. This unusual feature of the steady-state transitions has motivated many studies that involve developments of new methods^6–8^ and new models^9–12^ with an aim to understand nonequilibrium phase transitions, and to obtain the phase diagram in the parameter space of the problem.

The bulk-boundary duality implies the existence of multiple scales which result in stiff differential equations for the density profile. The difficulty lies in finding the nature of the density profile in any scale–the bulk or the boundary–consistent with the given boundary conditions. The usual procedure for problems with multiple scales is the boundary layer analysis^13–15^ which involves asymptotic matching of different parts of the solutions obtained for different scales. More specifically, the rapidly varying BL solution and the smoothly varying bulk solution must match in the overlapping asymptotic limits^15^. Such an approach, in an order-by-order scheme for separate scales, ultimately leads to nonphysical divergences, which need to be handled by a renormalization group (RG) analysis. We show here how RG determines the global density profile *across the scales*, with the BL as the starting point, thereby reinforcing BBD in a stronger form.

The power of the RG approach as a tool for asymptotic analysis has been illustrated in refs.^16–18^, for different types of nonlinear problems. We develop a procedure where the width of the BL is taken as the parameter to be renormalized to remove the divergences with the help of an arbitrary length scale *μ* that adjusts the location of the BL, and allows us to bridge the scales in the problem. The condition that the density profile should not depend on *μ*, then yields the RG equation for the width. The solution of the RG equation allows us to reconstruct the density profile. In short, once the long distance divergence is removed we get the entire density profie rather than solutions in parts, matched appropriately to generate a uniform solution. Thus obtaining the entire density profile from the renormalization of the boundary layer solution appears to be a clear manifestation of the bulk-boundary duality.

Let us consider TASEP with an additional adsorption and evaporation of particles to and from the lattice (Langmuir kinetics (LK))^9–12^. The dynamics of particles can be described through the time evolution of the occupancy variable, *τ*_*i*_, taking values 1 or 0 depending on whether the *i* th site is occupied or empty, respectively. The master equation for the statistically averaged occupancy variable, 〈*τ*_*i*_〉, is

1a $$\frac{d\langle {\tau }_{i}\rangle }{dt}=\langle {\tau }_{i-1}(1-{\tau }_{i})\rangle -\langle {\tau }_{i}(1-{\tau }_{i+1})\rangle +{\omega }_{a}\langle 1-{\tau }_{i}\rangle -{\omega }_{d}\langle {\tau }_{i}\rangle ,$$

where the first two terms on the right hand side of Eq. (1a) represent the hopping of particles to the empty forward site, and the last two terms represent adsorption and evaporation of particles with rates *ω*_*a*_ and *ω*_*d*_, respectively^19^. For a finite lattice of *N* sites, particles are injected at *i* = 1 at rate *α* and are withdrawn from the lattice at *i* = *N* at rate *β*. The time evolution of the average occupancy variable for the boundary sites can be written as

1b $$\frac{d\langle {\tau }_{1}\rangle }{dt}=\alpha \langle 1-{\tau }_{1}\rangle -\langle {\tau }_{1}(1-{\tau }_{2})\rangle ,$$

1c $$\frac{d\langle {\tau }_{N}\rangle }{dt}=-\beta \langle {\tau }_{N}\rangle +\langle {\tau }_{N-1}(1-{\tau }_{N})\rangle ,$$

where the *α*-, *β*- terms are the boundary terms, resembling the adsorption-evaporation terms in Eq. (1a). The boundary parameters *α*, *β* are crucial for the open chain problem because the steady state phase diagram is determined by these two parameters and the phase diagram is generally drawn in the *α*–*β* plane. Without hopping, the sites in the bulk would have a steady or equilibrium density ${\rho }_{{\rm{LK}}}={\omega }_{a}/({\omega }_{a}+{\omega }_{d}).$ With hopping, the boundary sites are maintained at densities *α* and 1 − *β*, for large *N*, while there is a net current of the order of *ρ*(1 − *ρ*) where *ρ* is the average density. On the other hand, there is a net deposit on the whole lattice of the order of *Nω*_*a*_(1 − *ρ*) − *Nω*_*d*_*ρ*. For this net adsorption not to overwhelm the current, it is required that *ω*_*a*_*N*, *ω*_*d*_*N* are O(1). In this limit, called the scaling limit^9^, the *α* – *β* phase diagram is drastically modified by the LK process.

In a mean-field approximation, factorizing the correlations as 〈*τ*_*i*_*τ*_*j*_〉 ≈ 〈*τ*_*i*_〉〈*τ*_*j*_〉, the steady-state density in the continuum limit (the lattice spacing, *a* → 0, and *N* → ∞ with *Na* = 1), can be described through the equation

2a $$\varepsilon \frac{{d}^{2}\rho }{d{x}^{2}}+(2\rho -1)\frac{d\rho }{dx}+{\rm{\Omega }}({\rho }_{{\rm{LK}}}-\rho )=0,$$

where *x* denotes the location along the lattice, *ρ*(*x*) = 〈*τ*_*i*_〉 is the average density at *x*, *ε* ( = 1/(2*N*)) is a small parameter, and

2b $${\rm{\Omega }}=({\omega }_{a}+{\omega }_{d})N,\,{\rm{and}}\,{\rho }_{{\rm{LK}}}=\frac{{\omega }_{a}}{{\omega }_{a}+{\omega }_{d}}.$$

The boundary conditions (BC) are

$$\rho (x=0)=\alpha ,\,{\rm{and}}\,\rho (x=1)=1-\beta =\gamma .$$

To obtain Eq. (2a), the neighboring densities are written as

2c $$\rho (x\pm a)=\rho (x)\pm a\frac{d\rho }{dx}+\frac{{a}^{2}}{2}\frac{{d}^{2}\rho }{d{x}^{2}}\mathrm{....}$$

Note that $\rho ={\rho }_{{\rm{LK}}}$ is a solution of Eq. (2a), but it does not satisfy the two BC’s.

The differential equation Eq. (2a) is singular due to the small prefactor *ε* in front of the highest order derivative. In the extreme limit, *ε* = 0, the equation reduces to a first order equation which cannot, in general, satisfy two BCs. The loss of one BC leads to the appearance of a boundary layer. Another way of seeing this is to realize that for small but finite *ε*, there are two scales, *x*, and $\tilde{x}=x/\varepsilon$, so that the density is a function of two widely different scales, making the equation stiff to solve. Standard numerical procedures with special continuation strategies^20^ fail to converge for small *ε*. To overcome this problem, the steady-state behaviour of this system has been studied using various methods such as domain wall theory, boundary layer analysis, numerical simulations, etc.^7,8,21^.

The bulk steady state phases are obtained from the mean-field equation, Eq. (2a), with *ε* = 0, and the result seems to agree with various other non-mean-field studies, especially numerical simulations^9,10^. In recent times, it has been realized that due to the bulk-boundary duality, the boundary layers (which in a broader sense include shocks) actually contain information for the bulk, and just focusing on the BLs would suffice for the phase diagram^8,13^. Although it is known that the mean-field BL differs from those seen in simulations, but still the basic mechanism that the BL controls the shape of the density profile remains valid. Mean-field theory, as a nonperturbative approach to any interacting system, provides the basic framework to develop an understanding of the system, and then build on it for more details. Examples are critical phenomena, polymers with excluded volume interaction and many others. In the present context, developing a global picture of the BL and the bulk even at the mean-field level, is a difficult task, and is generally done via singular perturbation theory which gets into trouble as we show in the next section. In this spirit, we try to develop a renormalization group based approach that handles the BL of the mean-field equation and extends the idea of the bulk-boundary duality in a general way.

The particle conserving TASEP (*ω*_*a*_ = *ω*_*d*_ = 0) can exist broadly in three phases which are low-density (LD) with *ρ* < 1/2, high-density (HD) with *ρ* > 1/2, and maximal current (*ρ* = 1/2) phases^3^. The LD-HD phase boundary is at *α* + *γ* = 1 with a linear density variation, *ρ*(*x*) = *α* + (*γ* − *α*)*x*. Numerical simulation shows that on the phase boundary, there is a coexistence of phases with *ρ* = *α* and *ρ* = *γ* with a shock anywhere on the lattice. This can be understood from the LK case in the limit of Ω → 0.

**Figure 1:**
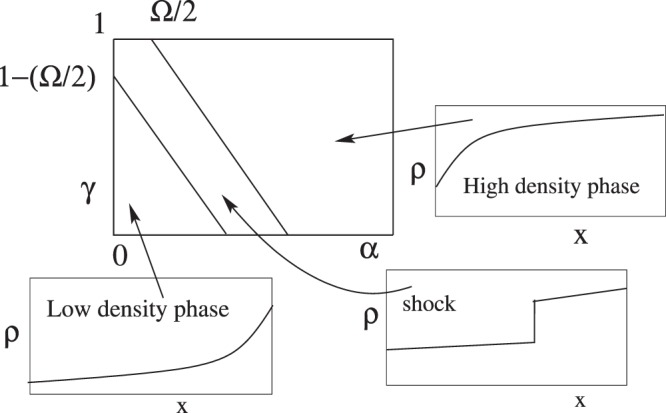
A part of the *γ*-*α* phase diagram (the region with *γ* ≈ 1) of TASEP with Langmuir kinetics with *ω*_*a*_ = *ω*_*d*_ (*r* = 0). The low-density phase to the shock phase boundary is *γ* = 1 − Ω/2 − *α*, while that for the shock to the high density phase is *γ* = 1 + Ω/2 − *α*. The density profiles in the three phases are shown schematically.

With LK, there is a difference in phase diagrams for *ρ*_LK_ = 1/2 and ${\rho }_{{\rm{LK}}}\ne 1/2$^9–11^. However, for both the cases, there is a region in the phase diagram where the high density (HD) and the low-density (LD) phases are separated by a shock phase. This region is of interest, see Fig. 1 corresponding to to $\gamma  > 0.5+\frac{{\rm{\Omega }}}{2}$. Unlike particle conserving TASEP, here the shock is localized with its location dependent on *α*, *γ*, *ω*_*a*_ and *ω*_*d*_. For *ρ*_LK_ = 1/2, the average particle density in the bulk changes linearly with *x*. In the LD phase for $\alpha  < \frac{1}{2}$, the average density across the lattice remains less than 1/2, consistent with the BC at *x* = 0, while a BL near *x* = 1 matches the BC at that end. Such a phase appears for $\alpha +\gamma  < 1-\frac{{\rm{\Omega }}}{2}$. Similarly, for *γ* > 1/2 and $\alpha +\gamma  > 1+\frac{{\rm{\Omega }}}{2}$, the system is in an HD phase in which the major part of the density is larger than 1/2, consistent with $\gamma  > \frac{1}{2}$ with the BL around *x* = 0. The picture remains more or less similar for ${\rho }_{{\rm{LK}}}\ne \mathrm{1/2}$ in this region, though the bulk density is nonlinear in *x*.

As Ω → 0, the shock region shown in Fig. 1, collapses onto the line *α* + *γ* = 1 as it should. As one moves across this shock phase by changing, say, *α*, the static shock position shifts from *x* = 0 to *x* = 1, no matter how small Ω is. By continuity, one therefore expects a shock separating the LD and HD phases to exist even on the *α* + *γ* = 1 line for Ω = 0 but the shock position remains labile. Assuming a uniform probability distribution of the shock position, the average density on this line becomes a linear one as mentioned above. That the features of the no-LK case can be revealed by the limit Ω → 0 justifies taking the LK case as the generic model for the TASEP class.

The RG analysis is based on the boundary layer part of the particle density profile, and the outcome is a globally valid solution for the entire density profile, thereby broadening the region of validity of the boundary layer solution^22^. We rewrite Eq. (2a) as

3a $$\frac{{d}^{2}\varphi }{d{\tilde{x}}^{2}}+\varphi \frac{d\varphi }{d\tilde{x}}+\varepsilon {\rm{\Omega }}(r-\varphi )=0,$$

where, $$\tilde{x}=x/\varepsilon ,$$

3b $$\varphi =2\rho -1,\,{\rm{and}}\,r=2{\rho }_{{\rm{LK}}}-1=\frac{{\omega }_{a}-{\omega }_{d}}{{\omega }_{a}+{\omega }_{d}}.$$

It is to be noted that Eq. (3a) remains invariant under a shift of origin $\tilde{x}\to \tilde{x}-\mu$; this symmetry is exploited below.

Let us look for a regular perturbative solution of the form,

4 $$\varphi ={\varphi }_{0}+\varepsilon {\varphi }_{1}+\ldots \mathrm{.}$$

The zeroth order solution is

5 $${\varphi }_{0}(\tilde{x})=p\,\tanh [\frac{p}{2}(\tilde{x}+k)],$$

which is characterized by two parameters *k* and *p*, related to the centre and the width of the boundary layer, respectively. In the boundary layer approach, this *ϕ*_0_ is the BL on the scale of $\tilde{x}$, to be matched with the bulk solution which can be found by solving Eq. (2a) for *ε* = 0 (see Supplementary Materials for details). We, instead, extend the BL solution to the next order. At $O(\varepsilon )$ level, *ϕ*_1_ satisfies the equation

6 $$\frac{{d}^{2}{\varphi }_{1}}{d{\tilde{x}}^{2}}+\frac{d({\varphi }_{0}{\varphi }_{1})}{d\tilde{x}}+{\rm{\Omega }}(r-{\varphi }_{0})=0.$$

The divergence mentioned earlier can now be seen from Eq. (6). It shows that ${\varphi }_{1}\sim \tilde{x}$, for $\tilde{x}\to \infty$, due to the limit *ϕ*_0_ → *p*.

The solution of Eq. (3a), *ϕ*, upto $O(\varepsilon )$, is given by

7 $$\varphi =p\,\psi (p,\tilde{x})-\varepsilon \frac{r-p}{p}{\rm{\Omega }}\,\tilde{x}\,\psi (p,\tilde{x})+\varepsilon  {\mathcal R} ,$$

8 $$\psi (p,\tilde{x})=\,\tanh (\frac{p}{2}\tilde{x}),$$

where only the diverging terms at the $O(\varepsilon )$ level are shown explicitly with $\varepsilon  {\mathcal R}$ representing all the regular terms together. See Supplementary Materials for details. This naive perturbation theory shows inconsistency as $\tilde{x}\to \infty$ since in this limit, the term at $O(\varepsilon )$ level in Eq. (8) becomes comparable to the zeroth order term. The divergence appearing in Eq. (8) can be isolated by introducing an arbitrary length scale *μ* that adjusts the location of the BL, to rewrite Eq. (8) as

9 $$\begin{array}{rcl}\varphi  & = & p\psi (p,\tilde{x})-\varepsilon \frac{r-p}{p}{\rm{\Omega }}(\tilde{x}-\mu )\psi (p,\tilde{x})\\  &  & -\,\varepsilon \mu \frac{r-p}{p}{\rm{\Omega }}\psi (p,\tilde{x})+\varepsilon  {\mathcal R} .\end{array}$$

By tuning *μ* we go from one scale to the other, with *μ* → ∞ approaching the bulk scale. Since *μ* is an arbitrary length-scale, it may be chosen in such way that $\tilde{x}-\mu$ is non-diverging, and, under such a scenario, the last term in Eq. (9) contains the divergence. In the following, we renormalize *p* to absorb this divergence in equation (9).

In the next step, we introduce a renormalized parameter *p*_*r*_ defined through the equation

10 $$p={p}_{r}(\mu )+\varepsilon {a}_{1}(\mu ),$$

to absorb the divergence in Eq. (9). Therefore, we set

11 $${a}_{1}=\frac{\mu }{{p}_{r}}(r-{p}_{r}){\rm{\Omega }}.$$

The divergence-free density profile now appears as

12 $$\begin{array}{rcl}\varphi  & = & {p}_{r}\psi ({p}_{r},\tilde{x})+\varepsilon \frac{\mu }{2}(r-{p}_{r})\frac{{\rm{\Omega }}\tilde{x}}{{\cosh }^{2}(\frac{{p}_{r}\tilde{x}}{2})}\\  &  & -\,\varepsilon \frac{r-{p}_{r}}{{p}_{r}}\psi ({p}_{r},\tilde{x})\,{\rm{\Omega }}\,(\tilde{x}-\mu ),\end{array}$$

where the cosh term comes from the Taylor expansion of *ψ*. This cosh term is a regular term since $1/cos{h}^{2}$ term decays exponentially for large $\tilde{x}$. Furthermore, since $\mu \sim \tilde{x}$, the last term in Eq. (12) is also divergence-free.

Since the final density profile must be independent of the arbitrary length scale *μ*, we must have ∂*ϕ*/∂*μ* = 0. The complete expression of ∂*ϕ*/∂*μ* along with cancellations necessary to ensure that ∂*ϕ*/∂*μ* is zero at $O(\varepsilon )$ level is shown in Supplementary Materials. This condition leads to the renormalisation group equation to $O(\varepsilon )$ as

13 $$\frac{d{p}_{r}}{d\mu }=-\,\varepsilon \,\frac{r-{p}_{r}}{{p}_{r}}\,{\rm{\Omega }}.$$

It is interesting to note that this RG equation is the bulk equation, Eq. (2a) with *ε* = 0, when expressed in terms of *ϕ*, and *μ* replacing *x*. Eq. (13) is analogous to the renormalisation group equation in the original formulation of Renormalization Group analysis^23^. In the present context, the importance of this equation lies in claiming that *ϕ* is independent of the arbitrary length *μ*. This equation can also be derived from the definition of *p*_*r*_(*μ*) in Eq. (10). But, as *p* appears as an arbitrary constant in Eq. (5), it is prudent to demand invariance of a physical quantity like *ϕ*.

For *r* = 0 (i.e., *ω*_*a*_ = *ω*_*d*_), the solution of Eq. (13) is *p*_*r*_ = Ω*εμ* + *c*, *c* being a constant. Substituting this in (12) along with $\mu =\tilde{x}$, we have the density profile, to leading order, *O*(*ε*^0^), as

14 $$\varphi (x)=(C+{\rm{\Omega }}x)\,\tanh \,[(C+{\rm{\Omega }}x)\,\tilde{x}/2],$$

where *C* and *x*_0_ (in $\tilde{x}=(x-{x}_{0})/\varepsilon$) are the constants to be fixed by the boundary conditions.

In case of *r* ≠ 0, we solve Eq. (13) perturbatively for small *r*. Expressing Eq. (13) in terms of *λ* = *ε*Ω*r*, we obtain a perturbative solution for *p*_*r*_ with ${p}_{r}={p}_{r}^{0}+\lambda {p}_{r}^{1}+O({\lambda }^{2})$ as

15 $${p}_{r}={\rm{\Omega }}\varepsilon \mu +c-r\,\mathrm{ln}({\rm{\Omega }}\varepsilon \mu +c)+O({\lambda }^{2}),$$

where *c* is a constant. Replacing *μ* by $\tilde{x}$, we have the final form of the density profile as

16 $$\begin{array}{rcl}\varphi  & = & [{\rm{\Omega }}x+C-r\,\mathrm{ln}({\rm{\Omega }}x+C)]\\  &  & \times \,\tanh \,[\{{\rm{\Omega }}x+C-r\,\mathrm{ln}({\rm{\Omega }}x+C)\}\,\tilde{x}/2].\end{array}$$

Eqs (14) and (16) are the main results of our paper. The bulk solutions can be found from these equations by considering $\tilde{x}\to \infty$ limit in which tanh → 1. In case of *r* = 0, *ϕ* approaches a linear function of *x* as obtained from the boundary layer analysis in ref.^10^. In case of *r* ≠ 0, the density profile *ϕ* in the $\tilde{x}\to \infty$ limit recovers the bulk solution which has a nonlinear dependence on *x*. The boundary layer parts, on the other hand, can be found from the $\tilde{x}\to 0$ limit of expressions in Eqs (14) and (16). As Eq. (14) shows, in the leading order, the density profile has a form $C\,\tanh (C\tilde{x}/2)$ in agreement with the results obtained through the boundary layer analysis^13,14^. Interestingly, the RG analysis, via the renormalization of the width because of adsorption/desorption kinetics of particles, leads to further subleading correction terms which contribute for finite *ε*. Instead of a simple additive form for the density over two scales, we see a more complex solution where the local bulk density affects the “local” width of the boundary layer. The *ε*-dependent term in Eq. (2a) comes from the diffusive contribution to the current, and therefore it is significant only in the region of rapid variation as in a BL or a shock. To leading order in the boundary layer analysis, this thin region does not generate much current from the Langmuir kinetics. As the BL thickens for not-so-small *ε*, there is an appreciable contribution from the Ω-dependent kinetics. Our RG analysis captures this aspect of the problem. Herein lies the importance of Eqs (14) and (16), which provide an interpolation formula from finite *ε* to the bulk.

**Figure 2:**
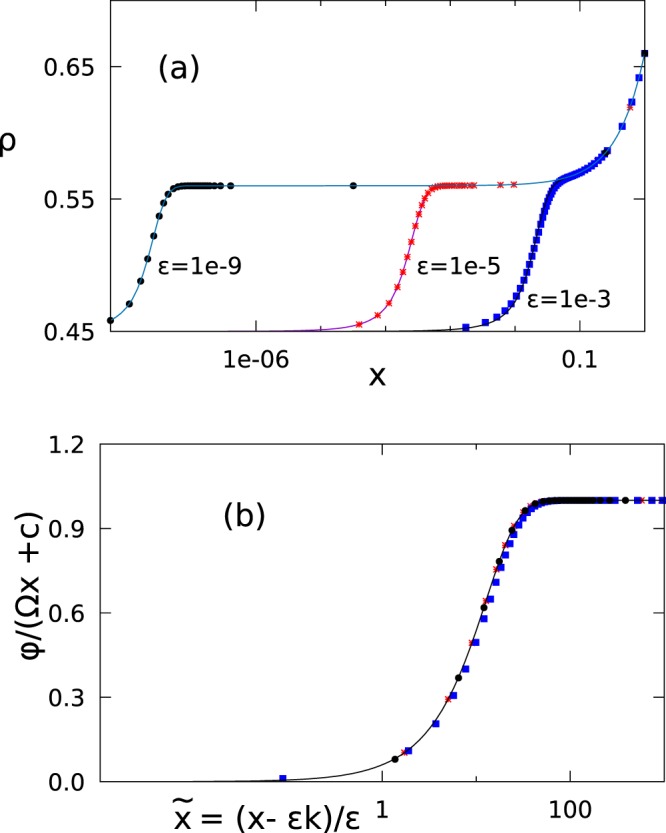
RG solution vs numerical results. (**a**) Data points are from numerical solutions of Eq. (2a) for three cases *ε* = 10^−3^, 10^−5^, 10^−9^ (as mentioned in the plot) with *r* = 0, Ω = 0.2, *α* = 0.45 and *γ* = 0.66. The RG solution of Eq. (14) are shown by solid lines. The value of *ε* in the asymptotic formula is adjusted to take care of higher order corrections. The values of *ε* for the solid curves are *ε* = 8.4 × 10^−4^, 10^−5^, 10^−9^. (**b**) Data collapse plot for the same set of data as in (**a**) (same symbols). Labels show the variables along x and y axes. The solid line is the tanh part of Eq. (14).

We compare the numerical solution of Eq. (2a) for *r* = 0, with plots obtained from the RG solution, Eq. (14). In Fig. 2, plots for the high-density phase with Ω = 0.2, and the boundary conditions *α* = 0.45 and *γ* = 0.66 are shown. The numerical solutions of the full differential equation for three different *ε*, viz., *ε* = 10^−3^, 10^−5^, 10^−9^ are shown here. For the RG solution in Eq. (14), the constants *C* and *x*_0_ = *εk* are found from the boundary conditions at *x* = 1 and *x* = 0. This is based on the observation that the boundary layer in the high density phase is formed at the *x* = 0 boundary. The equations are *C* + Ω = 0.32 and $C\,\tanh \,[C{x}_{0}\mathrm{/2}\varepsilon ]=0.1$, yielding *C* = 0.12 and *k* = 4.98. As mentioned in Fig. 2, the value of *ε* in the RG solution is adjusted to take care of the higher order corrections. Further, Eq. (14) admits a scaling form via a collapse of all curves for different *ε*’s if *ϕ*(*x*)/(*C* + Ω*x*) is taken as a function of $\tilde{x}=(x-{x}_{0})/\varepsilon$. Such a form is not expected from the naive boundary layer solution. A data collapse plot for all the numerical solutions is shown in Fig. 2b, confirming the predicted scaling.

**Figure 3:**
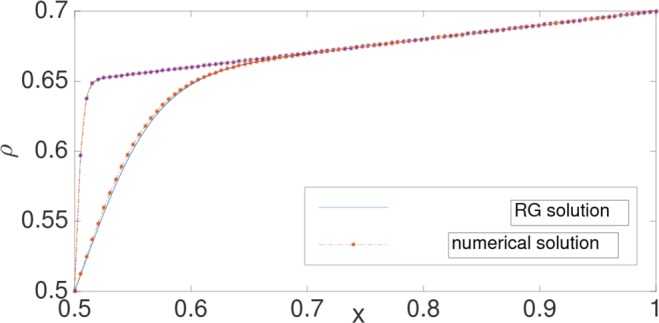
Density profile for shocks for *r* = 0, Ω = 0.2. The upper and lower curves correspond to *ε* = 0.001, and 0.01, respectively. The boundary parameters are *α* = 0.3 and *γ* = 0.7. The graph is plotted over the range *x* ∈ [0.5:1].

**Figure 4:**
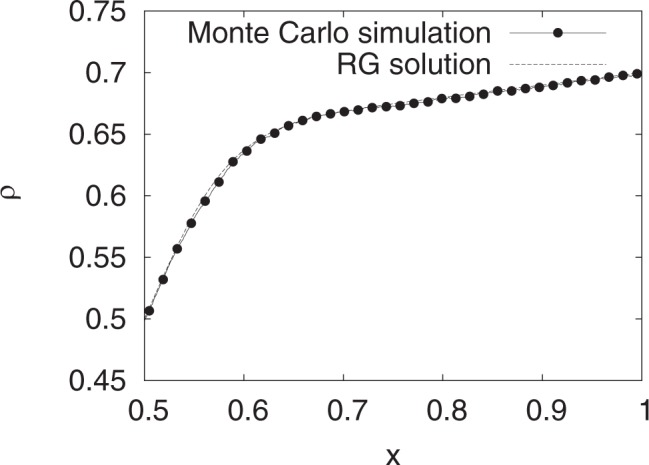
RG solution (dashed line) vs results from Monte Carlo simulations (line with dots). Density profile for shocks for *r* = 0, Ω = 0.2, *α* = 0.3 and *γ* = 0.7. Monte Carlo simulations were done for *N* = 1000 sites. For RG solution, 1/*ε* = 80 has been used.

We also compared the profiles for the shock phase. For a shock phase with *x*_0_ somewhere in the interior of the lattice, there is a symmetry $\varphi (\tilde{x})=-\varphi (-\tilde{x})$, obeyed by Eq. (3a). We, therefore, concentrate on $\tilde{x} > 0$ only. The boundary conditions chosen here are *α* = 0.3 and *γ* = 0.7, so that the shock is formed at *x*_0_ = 0.5. Eq. (2a) is solved numerically with these boundary conditions. With *x*_0_ = 0.5 and the BC at *x* = 1, we have *C* + Ω(*x* − *x*_0_) = 0.4, so that *C* = 0.3. The symmetry automatically fixes the boundary condition at *x* = 0. In this way, the RG analysis performed with boundary layer located near one of the boundaries can be utilized here. A good agreement is noted between the numerical solution of the full differential equation and the RG solution as given in Eq. (14) (see Fig. 3). Figure (4), shows a comparison of the RG result and the result from Monte Carlo simulation. For the RG result, *ε* is adjusted suitably to account for the higher order corrections.

In this paper, we developed a renormalization group scheme to determine the particle density profile in a one-dimensional, particle non-conserving totally asymmetric simple exclusion process. The particle adsorption/desorption kinetics (Langmuir kinetics) is the source of particle non-conservation while the steady state of nonzero current is maintained by the injection and the withdrawal rates at the boundaries. The continuum differential equation for the process is singular due to a small prefactor (*ε*) in front of its highest order derivative term that comes from diffusion. As a consequence of the singularity, the perturbative solution on the scale of $\tilde{x}=x/\varepsilon$ shows divergences at $O(\varepsilon )$ at large distances, $\tilde{x}\to \infty$. Upon absorbing the divergences systematically through renormalizations of the position and the width of the boundary layer, we arrive at a globally valid density-profile which describes both the boundary layer and its crossing over to the bulk solution. One of the predictions of the solution is the appearance of a finite-size scaling form for the density, which compares well with the results from direct numerical solutions of the steady-state differential equation in the high-density and the shock phases. We believe our procedure is general enough to apply to other boundary induced transitions as well.

## Supplementary information

LaTeX Supplementary File
